# Identification of Novel Natural Product Inhibitors against Matrix Metalloproteinase 9 Using Quantum Mechanical Fragment Molecular Orbital-Based Virtual Screening Methods

**DOI:** 10.3390/ijms23084438

**Published:** 2022-04-18

**Authors:** Hocheol Lim, Hansol Hong, Seonik Hwang, Song Ja Kim, Sung Yum Seo, Kyoung Tai No

**Affiliations:** 1 The Interdisciplinary Graduate Program in Integrative Biotechnology & Translational Medicine, Yonsei University, Incheon 21983, Korea; ihc0213@yonsei.ac.kr (H.L.); hshong@bmdrc.org (H.H.); 2 Bioinformatics and Molecular Design Research Center (BMDRC), Incheon 21983, Korea; 3 Department of Biotechnology, Yonsei University, Seoul 03722, Korea; sihwang@bmdrc.org; 4 Department of Biological Science, Kongju National University, Kongju 32588, Korea; ksj85@kongju.ac.kr (S.J.K.); dnalove@kongju.ac.kr (S.Y.S.); 5 Baobab AiBIO Co., Ltd., Incheon 21983, Korea

**Keywords:** matrix metalloproteinase 9, fragment molecular orbital, quantum chemistry, virtual screening, structure-based drug design, laetanine, genkwanin

## Abstract

Matrix metalloproteinases (MMPs) are calcium-dependent zinc-containing endopeptidases involved in multiple cellular processes. Among the MMP isoforms, MMP-9 regulates cancer invasion, rheumatoid arthritis, and osteoarthritis by degrading extracellular matrix proteins present in the tumor microenvironment and cartilage and promoting angiogenesis. Here, we identified two potent natural product inhibitors of the non-catalytic hemopexin domain of MMP-9 using a novel quantum mechanical fragment molecular orbital (FMO)-based virtual screening workflow. The workflow integrates qualitative pharmacophore modeling, quantitative binding affinity prediction, and a raw material search of natural product inhibitors with the BMDMS-NP library. In binding affinity prediction, we made a scoring function with the FMO method and applied the function to two protein targets (acetylcholinesterase and fibroblast growth factor 1 receptor) from DUD-E benchmark sets. In the two targets, the FMO method outperformed the Glide docking score and MM/PBSA methods. By applying this workflow to MMP-9, we proposed two potent natural product inhibitors (laetanine **9** and genkwanin **10**) that interact with hotspot residues of the hemopexin domain of MMP-9. Laetanine **9** and genkwanin **10** bind to MMP-9 with a dissociation constant (K_D_) of 21.6 and 0.614 μM, respectively. Overall, we present laetanine **9** and genkwanin **10** for MMP-9 and demonstrate that the novel FMO-based workflow with a quantum mechanical approach is promising to discover potent natural product inhibitors of MMP-9, satisfying the pharmacophore model and good binding affinity.

## 1. Introduction

Matrix metalloproteinases (MMPs) are calcium-dependent zinc-containing endopeptidases that degrade extracellular matrix proteins and participate in tissue remodeling and signaling events [1,2]. MMPs are involved in multiple cellular processes, including proliferation, migration, cancer invasion, host defense, angiogenesis, and metastasis [3,4]. Twenty-three MMPs have been identified in humans, including secreted and membrane-bound forms, sharing common structural and functional domains [2]. These 23 MMPs have multiple domains and typically include a signal sequence, propeptide, catalytic domain, linker domain, and hemopexin domain [5]. The catalytic domain of MMPs contains a zinc-ion-binding motif. Although many MMP inhibitors have been developed, they exhibit adverse effects in the clinical trial stage because of the lack of specificity between the target MMP and other members of the MMP family, as the zinc-binding site is common in all MMPs [6].

To overcome the adverse effects caused by MMP inhibitors targeting the zinc-binding site, the inhibition of the less conserved and non-catalytic domains of MMPs has been suggested to increase the specificity and selectivity [7,8]. Since the hemopexin domains are less conserved among MMPs, they have been considered a suitable site for the development of selective inhibitors of MMPs [7,8]. Moreover, among MMPs with the hemopexin domain, the gelatinase subfamily (MMP-2 and MMP-9) is important in collagen degradation through the digestion of gelatin, which is generated by collagenases [9]. MMP-9 is a 92 kDa type-IV collagenase that can degrade type-IV collagen and other extracellular matrices. MMP-9 is involved in tumor growth initiation and invasion in basal and squamous cell carcinomas and is associated with the radial growth phase of melanoma and tumor angiogenesis [4]. The expression levels of MMP-9 in patients with breast cancer are correlated with poor prognosis [7]. Therefore, suppressing MMP-9 via the hemopexin domain would be a good strategy for inhibiting MMP-9-mediated pathological processes.

Natural products have been considered an important source of lead and candidate compounds for new medicines in drug discovery because they provide efficient and wide coverage of drug-like chemical spaces [10]. Although natural products possess vast chemical diversity and are a rich source of novel compound classes for biological studies [11], challenges related to the quality control of materials and commercial availability exist. Recently, Lee et al. developed the Bioinformatics and Molecular Design Research Center Mass Spectral Library—Natural Products (BMDMS-NP), which was sufficiently exhaustive to represent the structural diversity of commercially available natural products [12]. Therefore, to the best of our knowledge, the BMDMS-NP library is a good starting point for the discovery of natural product inhibitors.

Structure-based drug design (SBDD) is considered one of the most powerful tools for drug discovery [13]. The necessary pharmacological activities of drugs are based on their three-dimensional structures. Understanding their complex structures is critical in SBDD methods. Since the requirement for the therapeutic effect of a ligand is the capability of the ligand to bind to the target protein, the systematic analysis of the binding complex between the target protein and the ligand through SBDD is a crucial step in drug discovery. The binding affinity of a ligand for a given target protein can be calculated from the binding free energy between the ligand and target protein. Therefore, to explain the difference in binding affinity obtained from the experiment, the energies calculated in the SBDD must also correspond to the free energy.

Structure-based virtual screening (SBVS) methods have reduced the number of unnecessary experiments, which has led to a decrease in the time and cost involved in the lead compound discovery. Most scoring functions in SBVS methods are rooted in free energy calculations with a molecular mechanical empirical potential energy function (a force field) [14] or quantum mechanical (QM) molecular orbital calculations, allowing the estimation of changes in the binding affinity between the target protein and ligands. Empirical potential energy functions have frequently been used to study protein–ligand complexes in SBVS methods because of their low computational cost [15]. For example, docking scores and the molecular mechanics/generalized Born surface area (MM/GBSA) method are generally used to predict the binding affinities of small molecular ligands [16,17,18,19]. However, the empirical potential energy functions in a force field are adopted to reproduce the mean energy and lack many important effects, such as electronic polarization, charge transfer, and halogen bonding [15], which leads to the imprecise description of protein–ligand complexes. The use of QM methods as scoring functions is promising because it can help explain important effects while hindering many applications of the QM method to the SBVS process for large biological systems due to the high computational cost.

Fragment molecular orbital (FMO) was developed by Kitaura et al. in 1999 and offers faster computational speeds than the traditional QM method without the loss of accuracy due to the fragmentation of biological systems [20]. The FMO method and pair interaction energy decomposition analysis (PIEDA) have been used to analyze protein–ligand interactions and provide accurate information for investigating the binding potential [21,22]. Even with the FMO method, applications to SBVS still incur high computational costs. Recently, the density-functional tight-binding (DFTB) and polarizable continuum model (PCM) methods were combined with the FMO method [23]. FMO–DFTB/PCM showed efficient predictions of protein–ligand interactions with moderate accuracy and a fast computational speed [24]. Morao et al. demonstrated that FMO–DFTB3/PCM showed good correlations with the experimental values of pKi for human β2-adrenoceptor ($R^{2}=0.7833$), and pIC50 for human P2Y12 receptor ($R^{2}=0.8121$) [24]. The FMO–DFTB method outperformed the GBVI/WSA force-field-based scoring function in identifying a set of 10 binders and 500 decoys of the human k-opioid receptor [25]. Therefore, FMO–DFTB/PCM has the potential to act as a scoring function in the SBVS method.

In this study, we devised a novel quantitative FMO-based virtual screening workflow to identify novel natural product inhibitors of the MMP-9 enzyme. To validate the FMO-based scoring function in the workflow, we applied the method to two protein targets (acetylcholinesterase and fibroblast growth factor 1 receptor) from DUD-E benchmark sets [26] and compared the performance using the Glide docking score and MM/PBSA methods. We applied the newly devised workflow to MMP-9 in five steps. First, to develop a modified pharmacophore model and obtain hotspot information, we performed high-level FMO–RIMP2/PCM calculations with the complex structures between the reference ligands and MMP-9 from molecular docking simulations. Second, we performed pharmacophore-based virtual screening using the BMDMS-NP library and selected the first virtual hits. Third, we performed molecular docking followed by FMO analysis at the FMO–DFTB3/D/PCM level with the first virtual hits and selected the second virtual hits based on the hotspot interaction profiles and ranking using the FMO scoring function. Fourth, we performed gelatin zymography assays with the first hits, repeated the third step at the high-level FMO–RIMP2/PCM level, and finally selected the top two hits. Fifth, we performed a surface plasmon resonance (SPR) analysis to measure the binding affinities of the top hits with MMP-9. Finally, we searched the raw materials of the two hits using the integrated database and found a correlation between the reported physiological effects of the raw materials and MMP-9. As a result, we identified the two natural product inhibitors with the novel FMO-based virtual screening workflow and demonstrated that this workflow could be used as a promising strategy to discover novel natural product inhibitors of MMP-9.

## 2. Materials and Methods

### 2.1. Structure Preparation

The X-ray structures of acetylcholinesterase (PDB ID: 1E66) and fibroblast growth factor receptor 1 (PDB ID: 3C4F) were retrieved from the Protein Data Bank [27,28,29]. All missing side chains and loops of the protein were filled using Prime implemented in the Schrödinger suite (ver. 2018-3) [30,31]. Hydrogen atoms were added to the structure at pH 7.0, and their positions were optimized using the PROPKA module implemented in the Schrödinger suite (ver. 2018-3) [32]. Restrained energy minimization was performed on the structure with OPLS3 within 0.3 Å root-mean-square deviation [33]. Ligand structures of the actives and decoys of each target were prepared using the LigPrep module implemented in the Schrödinger suite (ver. 2018-3) [34]. A total of 446 actives and 484 experimental decoys were used for acetylcholinesterase, and 225 actives and 143 experimental decoys were used for fibroblast growth factor receptor 1. Duplicate structures were removed from each benchmarking set. The X-ray crystal structure of the hemopexin domain of MMP-9 was retrieved from the Protein Data Bank (PDB ID: 1ITV). The protein structure and six known inhibitors (**1–6**) of the hemopexin domain of MMP-9 were prepared in the same way. All inhibitors of MMP-9 in this study are shown in Supplementary Figure S1.

### 2.2. Pharmacophore-Based Virtual Screening

Pharmacophore-based virtual screening was conducted using the Phase module implemented in the Schrödinger suite (ver. 2018-3) from the BMDMS-NP library [35,36]. The 3D pharmacophore model of MMP-9 has eight features: two hydrogen acceptors, three hydrogen donors, one positive ionic feature, one hydrophobic feature, and one aromatic ring. In the pharmacophore model, we treated hydrophobic and aromatic ring features as equivalent and treated acceptor and negative features as equivalent. The first 467 virtual hits were selected from 2739 natural products in the BMDMS-NP library.

### 2.3. Molecular Docking

Molecular docking simulations were performed using the Glide-SP in the Prime module, implemented in the Schrödinger suite (ver. 2018-3) [17]. In the Glide-SP protocol, 5000 conformers per ligand were generated and maintained for energy minimization during the initial phase of docking. To utilize Glide, which was designed as close to an exhaustive search [17], we generated all possible poses and performed post-docking minimization for all generated poses. The top 10 docking poses of each virtual hit were selected by ranking the docking poses with Emodel scores and removing duplicate docking poses by visual inspection [18].

### 2.4. MM-GBSA Simulation

Molecular mechanics generalized Born surface area (MM-GBSA) simulations were performed using the Prime module implemented in the Schrödinger suite (ver. 2018-3) [37]. MM-GBSA simulation is one of the most popular methods for estimating the relative binding affinities of protein–ligand complexes [16]. The relative binding free energy, $\Delta G_{bind}$, was estimated using Equation (1):

(1) $$\Delta G_{bind}=G_{complex}-G_{protein}-G_{ligand}$$

where $\Delta G_{bind}$ represents the difference between the energy of the protein–ligand complex state in an aqueous solution and the energy of the states in which the ligand and protein do not interact in an aqueous solution. The brackets indicate ensemble averages. $G_{complex}$ is the average free energy in the complex calculated using the MM-GBSA method, whereas $G_{protein}$ and $G_{ligand}$ are the energies in the unbound protein and ligand after separating the protein from the complex.

### 2.5. Fragment Molecular Orbitals (FMOs)

Fragment molecular orbital is a linear-scaling QM method that transforms a larger system into smaller parts called fragments. By performing QM calculations on the fragments, the FMO method dramatically reduces the computational cost. The two-body FMO calculation consists of four consecutive processes: fragmentation, fragment self-consistent field (SCF) calculation, fragment pair SCF calculation, and evaluation of total properties such as energy and gradient [38,39].

In the fragmentation step, we used one-fragment-per-residue fragmentation, where a ligand or each residue of a target protein was defined as a fragment. The residues were divided into fragments at the alpha carbon sites based on hybrid projection orbital scheme fragmentation to decrease the computational cost and correct errors from the projection operator [40]. Two cysteine residues that formed disulfide bonds in the target protein were defined as one fragment. All molecular orbitals of each fragment (monomer) were optimized in the electrostatic field of the entire system by SCF cycles, while the energies and electron densities were optimized simultaneously [39]. The same process was then applied to the fragment pair (dimer). The difference between the process of the monomer and that of the dimer is the size of the fragment, because the two fragments in the monomer are treated as a fragment in the dimer. The energies of the monomers and dimers were used to determine the total energy of the system. Especially in drug discovery, the binding affinity of the protein–ligand system is simply approximated as the sum of the PIE. The PIE in the FMO calculations was defined using Equation (2) and was decomposed using PIEDA to provide more physical details [41].

(2) $$\Delta E_{\mathrm{ij}}^{int}=\Delta E_{ij}^{ES}+\Delta E_{ij}^{EX}+\Delta E_{\mathrm{ij}}^{CT+mix}+\Delta E_{ij}^{DI}+\Delta G_{sol}$$

The PIE ($\Delta E_{\mathrm{ij}}^{int}$) between fragments i and j is composed of five energy terms: electrostatic ($\Delta E_{ij}^{ES}$), exchange-repulsion ($\Delta E_{ij}^{EX}$), charge transfer with the higher-order mixed term ($\Delta E_{\mathrm{ij}}^{CT+mix}$), dispersion ($\Delta E_{ij}^{DI}$), and solvation energy ($\Delta G_{sol}$). The binding affinities between the target protein and ligand were approximated using the TIE, which is the sum of PIEs between the target protein and ligands. It is important to note that TIE is not the difference between the energies of the free and bound ligands, but rather indicates the strength of the interactions between the target protein and the ligand [42].

All ab initio FMO calculations were performed using the 30 June 2019 version of GAMESS software [43]. To investigate key interactions, the two-body FMO method was applied to 10 complexes between MMP-9 and 10 ligands (six reference ligands and four hits) at the resolution of the identity second-order Møller–Plesset perturbation (RIMP2) [44] and polarizable continuum model (PCM) [45] with the 6–31 G** basis set (FMO–RIMP2/PCM level). The two-body FMO method was used at the FMO–DFTB3/D/PCM level to rank the docked ligands. An extension of the self-consistent charge-density-functional tight-binding method is derived via a third-order expansion of the density functional theory method (DFTB3) [46]. DFTB3 calculations were performed with the 3OB parameter set [47], UFF-type dispersion correction (DFTB3/D) [48,49], and polarizable continuum model (PCM) [23]. Based on the previous reports [21,38,42,50], we considered the interaction with a pair interaction energy more stable than −3.0 kcal/mol to be significant. In the FMO calculations, we included the entire hemopexin domain from the crystal structure.

### 2.6. FMO-Based Virtual Screening

FMO-based virtual screening consists of three steps. First, we performed FMO/PIEDA analysis with known protein–ligand complexes at the FMO–RIMP2/PCM level. In the FMO/PIEDA analysis, we quantitatively identified the hotspot residues, which have significant interactions that are more stable than −3.0 kcal/mol with at least half of the reference ligands. In the second step, we generated a pharmacophore model using the hotspot information obtained in the first step. We then performed pharmacophore-based virtual screening with the reference ligands for validation and the BMDMS-NP library for the first virtual hits. In the third step, we generated protein–ligand complex structures using molecular docking simulations and performed FMO analysis of the first virtual hit complexes at the FMO–DFTB3/D/PCM level to predict the binding affinity and investigate the hotspot interaction profile of each virtual hit. The binding affinities were approximated using the TIE calculated by the sum of the PIEs between the protein target and ligand. The hotspot interaction profiles are calculated by the sum of PIEs between the hotspot residues and ligand, and the hotspot similarity between virtual hits and reference ligands is measured in Tanimoto coefficients between the hotspot interaction profiles [51], where the value is 1 if PIE between the hotspot residue and ligand is more stable than −3 kcal/mol. The calculated binding affinities and hotspot profile similarities were used as the scoring functions to rank the first virtual hits. To evaluate the scoring function using FMO–TIE, similarity ranking was not applied to the two benchmarking sets. For MMP-9, we selected the virtual hits if the similarity score was more than 0.5, with at least one reference ligand, and ranked the filtered virtual hits with the ranking by TIE.

### 2.7. Gelatin Zymography and Wound-Healing Assays

Gelatin zymography assays were conducted as follows, based on the protocol described by Toth et al. [52]. HT1080, a human fibrosarcoma cell line, was cultured in Dulbecco’s modified Eagle’s medium (DMEM; Gibco-BRL, Grand Island, NY, USA) containing 10% fetal bovine serum (FBS; WelGENE, Daegu, Republic of Korea) and 1% penicillin and streptomycin (Sigma-Aldrich, Steinheim, Germany). In 100 mm cell culture dishes, HT1080 cells were seeded ($4\times 10^{5}$ cells/dish) and incubated for 24 h in a 5% CO2 incubator at 37 °C. After 24 h, the medium was changed to a 7 mL serum-free medium to obtain a more concentrated supernatant containing MMP-9. After incubation for 48 h, the medium was collected, and the supernatant was obtained after centrifugation at 10,000 rpm for 10 min. Stock solutions of hit compounds were dissolved in 100% DMSO at a concentration of 20 mM. Supernatants (35 uL) containing 300 μM of hit compounds were incubated for 1 h at 37 °C and loaded onto 7.5% SDS-polyacrylamide gels containing 0.1% gelatin. The gels were electrophoresed at 120 V and washed twice with 2.5% Triton X-100 for 90 min at room temperature. Subsequently, the gels were incubated in incubation buffer (10 mM CaCl2, 150 mM NaCl, and 50 mM Tris-HCl, pH 7.5) for 20 h. The gels were then stained using Coomassie Brilliant Blue solution and scanned using an LAS-3000 imager (Fuji film Co., Tokyo, Japan). Images of the gels were analyzed using ImageJ software (NIH, Bethesda, MD, USA).

Wound-healing assays were conducted as follows, based on the procedures of Rodriguez et al. [53]. Human A375SM cells were used in the wound-healing assays, because it was demonstrated that MMP-9 can be a marker of aggressiveness in several tumors, including melanoma [54,55]. Human A375SM cells were cultured in DMEM (Gibco-BRL, Grand Island, NY, USA) containing 10% FBS (WelGENE, Daegu, Republic of Korea) and 1% penicillin and streptomycin (Sigma-Aldrich, Steinheim, Germany), seeded in 35-mm cell culture dishes ($2\times 10^{5}$ cells/dish), and incubated for 24 h. After 24 h, using a 200 μL pipette tip, the cell monolayers were scratched to make a straight-line scratch and then washed with a medium to remove cell debris. The hit compound was dissolved in 100% dimethyl sulfoxide (DMSO) at a concentration of 20 mM. After washing, the cells were treated with 30 μM of hit compounds and incubated for 24 h. The same concentration of DMSO was used in the control group. The scratched areas were calculated using the TScratch program (CSElab) [56].

Each experiment was repeated three times and the data are presented as the means ± standard error of the mean. Statistical differences between groups were analyzed by the Student’s *t*-test. A *p*-value <0.05 was considered to indicate a statistically significant difference.

### 2.8. Surface Plasmon Resonance (SPR) Analysis

Purified recombinant human MMP-9 protein with a His-tag was prepared under catalog number 10327-H08H (Leehyo Bioscience Co., Ltd., Seongnam, Republic of Korea). The interactions between MMP-9 and natural product inhibitors (**9** and **10**) were investigated using Biacore 3000. Purified MMP-9 was immobilized on the HC1000M sensor chip with amine coupling with the running buffer (10 mM HEPES, 150 mM NaCl, and 0.005% Tweens20) at pH 7.4 and a flow rate of 10 μL/min. The ligands were diluted with the running buffer (10 mM HEPES, 150 mM NaCl, and 0.005% Tweens20) at pH 7.4 and 5% DMSO at eight different concentrations. Compound **9** was diluted with 0, 6.25, 12.5, 25, 50, 100, 200, and 400 μΜ; compound **10** was diluted with 0, 0.78, 1.56, 3.13, 6.25, 12.5, 25, and 50 μΜ. Ligands were passed over the chip at a flow rate of 30 μL/min. Regeneration was performed with 0.5 M NaCl at a flow rate of 10 μL/min. The association constant ($k_{a}$ in $M^{-1}s^{-1}$) represents the rate of complex formation, and the dissociation rate constant ($k_{d}$ in $s^{-1}$) represents the rate of complex decay. High binding affinity interactions were characterized by low $K_{D}$ values, which were represented by the equation $K_{D}=k_{d}/k_{a}$.

### 2.9. Search of Raw Materials with Integrated Database

A raw material information search was conducted using an in-house, integrated database. The resources of the integrated database were downloaded from COCONUT, FooDB, and KNApSAcK databases [57,58,59]. These resources include information on metabolites, plant species, and metadata from functional products and diseases. Plant species information was retrieved from the names and structures of the final hits.

## 3. Results

**Figure 1:**
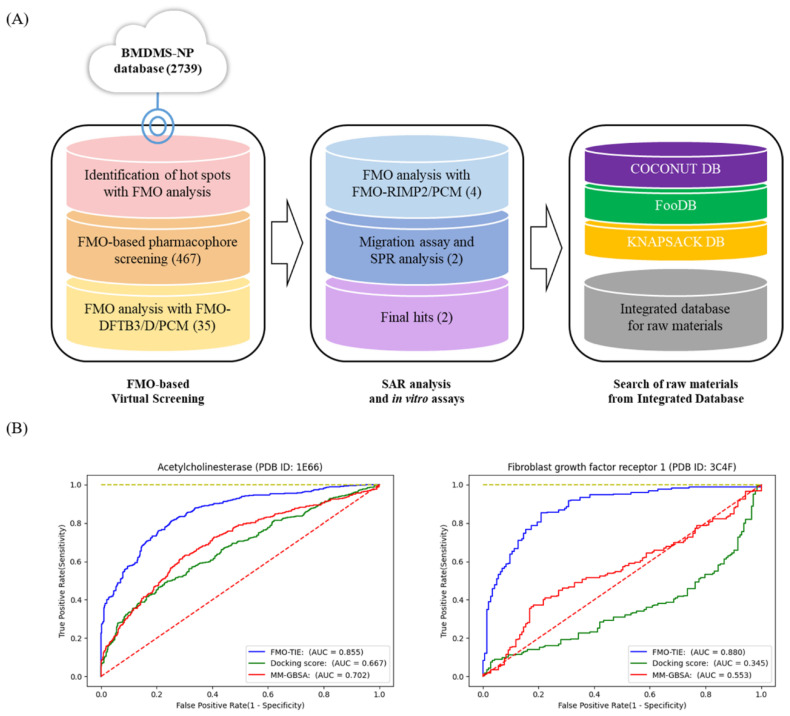
FMO-based workflow and scoring function evaluation in FMO-based virtual screening. (**A**) FMO-based workflow to discover natural product inhibitors of MMP-9 and find their respective raw material information. (**B**) Evaluation of the scoring function in FMO-based virtual screening in the two benchmarking sets.

To find novel natural product inhibitors targeting the hemopexin domain of MMP-9, we devised an FMO-based workflow by integrating qualitative pharmacophore modeling, quantitative binding affinity prediction, and raw material search of hit compounds. The workflow in this study is shown in Figure 1A and consists of three steps: FMO-based virtual screening, structure–activity relationship (SAR) analysis, SPR analysis, and the search for raw materials obtained from an integrated database.

### 3.1. FMO-Based Virtual Screening and Validation with Two Benchmarking Sets

The FMO method enables us to quantify the molecular interactions between ligands and protein residues and even the interactions between protein residues. FMO-based virtual screening includes three steps: the identification of hotspots by FMO analysis, FMO-based pharmacophore screening, and FMO analysis with FMO–DFTB3/D/PCM.

First, to identify the hotspot residues between the target protein and the known small molecular inhibitors, we performed a high-order FMO–RIMP2/PCM analysis of the reference complexes. The hotspot residues were identified with the common significant interactions in the known small molecular inhibitors based on the FMO results, where we defined the interactions present in half of the reference ligands as common. Second, we made a pharmacophore model with the known small molecular inhibitors and modified the model with the hotspot residue information not only for the shape of the known small molecular inhibitors but also for the interactions with the target protein. Third, to accurately quantify the binding affinities of the ligands, we applied FMO analysis as a scoring function in the SBVS method. Because the QM method majorly depends on structural coordinates, we generated 5000 conformers per ligand and performed molecular docking and post-docking minimization with all generated conformers to find plausible binding poses. Because Glide approximates a complete systematic search of the conformational, orientational, and positional space of the docked ligand [17], we assumed that generating as many conformers as possible would increase the possibility of generating correct binding poses. Because the FMO calculations of all binding poses complexed with the target protein require high computational costs, we applied the pharmacophore models to reduce the costs. We selected the top 10 plausible binding poses per virtual hit based on the Emodel score to reduce computational cost again, having a relatively significant weighting of electrostatic and van der Waals interaction energies [17]. We then analyzed the top 10 binding poses by FMO analysis at the FMO–DFTB3/D/PCM level and ranked all virtual hits with total interaction energy (FMO–TIE) scores.

To evaluate the scoring function of the FMO analysis at the FMO–DFTB3/D/PCM level, we applied this method to two known protein targets (acetylcholinesterase and fibroblast growth factor receptor 1) from the DUD-E benchmarking set [26]. We selected two protein targets because the two sets had a balanced number of actives and experimental decoys. A receiver operating characteristic (ROC) curve of our method is shown as FMO–TIE in Figure 1B. We also showed the ROC curves from other scoring functions, i.e., from the Glide docking score and MM-GBSA simulation, where we selected the top score of the scoring functions in the top 10 binding poses. For acetylcholinesterase, the area under the ROC curve (AUC) of FMO–TIE was 0.855, whereas the AUCs of the docking score and MM-GBSA were 0.667 and 0.702, respectively. For fibroblast growth factor receptor 1, the AUC of FMO–TIE was 0.880, whereas those of the docking score and MM-GBSA were 0.345 and 0.553, respectively. The scoring function of the FMO analysis at the FMO–DFTB/D/PCM level outperformed the scoring functions from the Glide docking score and the MM-GBSA simulation in the two benchmarking sets.

### 3.2. Application of FMO-Based Virtual Screening to MMP-9

To discover specific natural product inhibitors of MMP-9, we applied a novel FMO-based virtual screening workflow to the hemopexin domain of MMP-9 using the BMDMS-NP library. Because the BMDMS-NP library is as structurally diverse as phytochemicals in the ZINC15 database [12], we used the BMDMS-NP library for the chemical library (Figure 1A).

**Figure 2:**
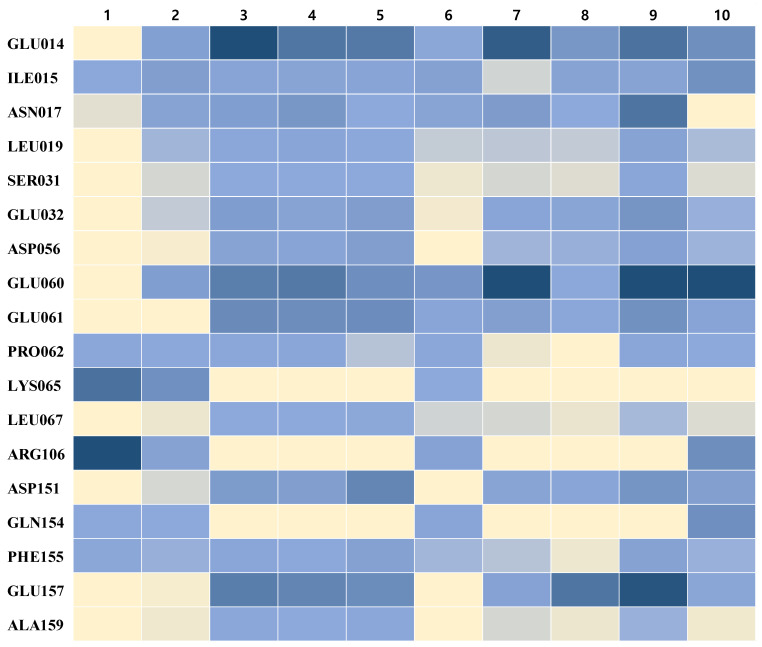
Comparison of hotspot residues of MMP-9 involved in ligand binding. Hotspot residues are represented in the rows. Ligands are represented in the columns. In each box of the matrix, interactions between the ligand and the residues are colored from dark blue (PIE < −100 kcal/mol) and blue (PIE < −3 kcal/mol), to light-yellow (PIE > 0 kcal/mol). The PIE values are calculated at the FMO–RIMP2/PCM level.

To prepare reference complexes with the hemopexin domain of MMP-9, we collected six inhibitors known to bind to the hemopexin domain [7,60] and created the complex structures using molecular docking simulations. To identify and investigate the hotspot residues of the hemopexin domain of MMP-9, we analyzed six reference complexes using high-level FMO analysis at the FMO–RIMP2/PCM level. The FMO results from the six ligands are shown in Supplementary Figures S2 and S3, which revealed 33 significant interactions between the six ligands and the hemopexin of MMP-9. We identified 18 hotspot residues, having significant interactions with at least three of six ligands that were more stable than −3.0 kcal/mol. The interaction map is shown in Figure 2. These results were in agreement with those of a previous study showing that Glu14, Glu60, Lys65, Arg106, and Gln154 residues are essential in the hemopexin binding pocket [61]. The interaction map showed two different hotspot patterns for the six ligands. Compounds **1** and **2** showed one pattern of interactions with Lys65, Arg106, and Gln154, whereas compounds **3**, **4**, and **5** showed the other pattern of interactions with Glu14, Ser31, Glu32, Asp56, Glu60, Glu61, Leu67, Asp151, Glu157, and Ala159. Moreover, compound **6** showed a mixed pattern of interactions with Glu14, Lys65, Arg106, and Gln154.

Based on the two interaction patterns from the FMO analysis and the shape of the six reference structures, we generated a modified pharmacophore model with eight features for the hemopexin domain and illustrated the model in Supplementary Figure S4. To validate the pharmacophore model, we applied the model to six ligands and obtained the six ligands with actives after setting the criteria of at least four of the eight features. We then performed pharmacophore-based virtual screening with the BMDMS-NP library and obtained the first 467 virtual hits.

To predict the binding affinities of the first virtual hits, we performed an FMO analysis at the FMO–DFTB3/D/PCM level. We generated all plausible binding poses with Glide by performing molecular docking and post-docking minimization with all generated conformers. We obtained the top 10 binding poses per virtual hit with the Emodel score and analyzed the top 10 complexes with FMO analysis at the FMO–DFTB3/D/PCM level. We then ranked the virtual hits by FMO–TIE and additionally checked whether the hotspot profiles of the virtual hits had a Tanimoto similarity of more than 0.5 with six ligands. Finally, we selected the first 35 virtual hits and performed gelatin zymography. From gelatin zymography assays, we selected the top four hits (**7**, **8**, **9**, and **10**) from the first virtual hits (Supplementary Figure S5).

### 3.3. Novel Ligands Targeting the Hemopexin Domain of MMP-9

**Figure 3:**
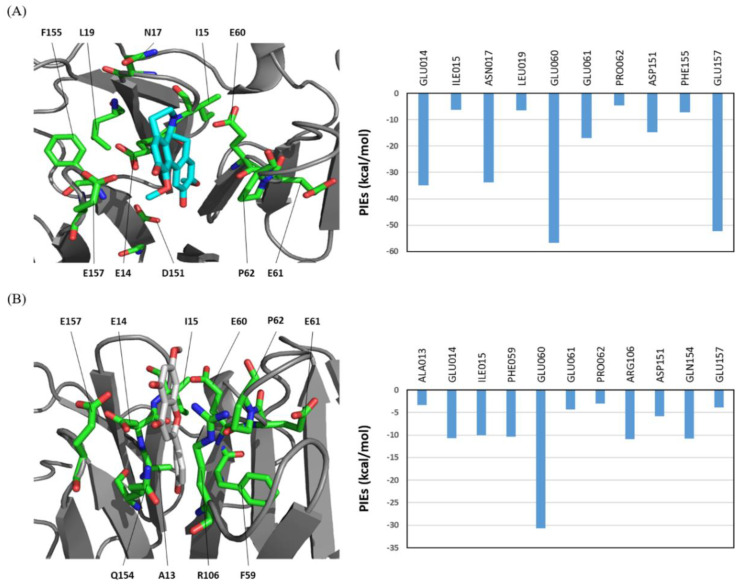
The FMO results for compounds 9 and 10 in complex with the hemopexin domain of MMP-9. (**A**) The structure of compound **9** binding to the hemopexin domain of MMP-9. The carbon atoms of **9** are shown in cyan. (**B**) The structure of compound **10** binding to the hemopexin domain of MMP-9. The carbon atoms of **10** are shown in white. The carbon atoms of the residues of MMP-9 are shown in green. The nitrogen and oxygen atoms are shown in blue and red, respectively. The right bar plot describes the PIEs of the significant residues in the hemopexin domain of MMP-9. All interactions shown here have attractive PIE values more stable than −3.0 kcal/mol. The PIE values are calculated at the FMO–RIMP2/PCM level.

To more accurately investigate the hotspot interaction profiles between MMP-9 and the top four hits, we performed FMO analysis with high-level FMO–RIMP2/PCM calculations and illustrated the FMO results in Figure 3 and Supplementary Figure S6. The top four second virtual hits had similar hotspot interaction patterns to those of the six ligands shown in Figure 2, and their Tanimoto similarities are shown in Supplementary Figure S7. The FMO analysis detected seven significant interactions in compound **7** and eight significant interactions in compound **8**. They had a partial hotspot interaction pattern compared with those of the six ligands, in which the similarity values of compounds **7** and **8** with compound **5** were 0.5 and 0.57, respectively. In contrast, FMO analysis detected nine significant interactions between compound **9** and nine residues of MMP-9: Glu14, Ile15, Asn17, Glu60, Glu61, Pro62, Asp151, Phe155, and Glu157. Moreover, FMO analysis detected 11 significant interactions between compound **10** and 11 residues of MMP-9: Ala13, Glu14, Ile15, Phe59, Glu60, Glu61, Pro62, Arg106, Asp151, Gln154, and Glu157. Compound **9** had a similar hotspot interaction pattern to compounds **3**, **4**, and **5**, with similarity values of 0.867, 0.867, and 0.8, respectively. Compound **10** had a mixed hotspot interaction pattern, like that of compound **6**, in which the similarity value was 0.64.

**Figure 4:**
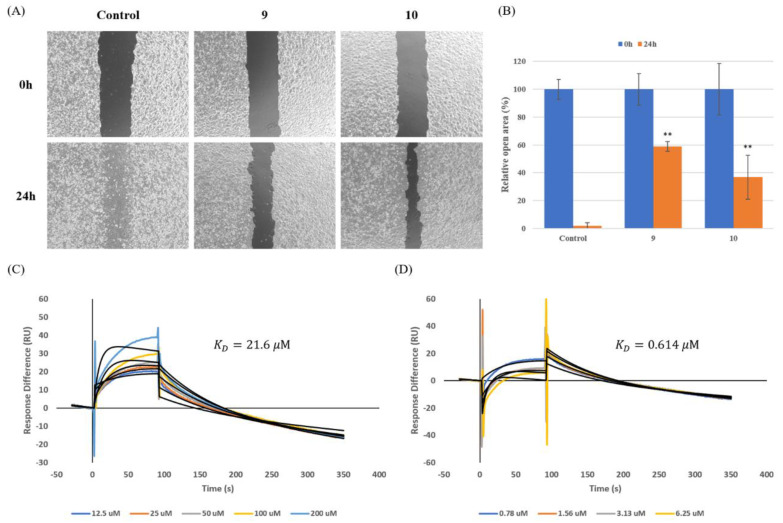
The inhibition of MMP-9 by compounds 9 and 10. (**A**) Wound-healing assays for A375SM cells exposed to 30 μM for 24 h. The wounded region was observed with the TScratch program. (**B**) The results of the wound-healing assays. Columns are the mean percentage of the relative open area after 24 h compared to 0 h, and the error bars are the standard error of the mean (*n* = 3). ** *p* < 0.01 vs. control group. (**C**) Sensorgrams of **9** binding to MMP-9. (**D**) Sensorgrams of **10** binding to MMP-9.

Based on the FMO results, we selected the top two hits and performed a wound-healing assay and SPR analysis, as illustrated in Figure 4. Since extracellular matrices must be degraded by MMPs for cell migration, a wound-healing assay can show the inhibition of MMPs. In the wound-healing assays, the A375SM cells treated with compound **9** or **10** showed lower migration rates than the control (59 and 36.8% of the relative open area, respectively). SPR analysis was performed to determine whether the top two compounds (**9** and **10**) directly bind to MMP-9. We measured the binding affinity between MMP-9 and the top two hits (Figure 4). Compound **9** exhibited a dose-dependent change with a dissociation constant ($K_{D}$) of 21.6 μM, where the $k_{a}$ was $5.79\times 10^{2}{\;M}^{-1}s^{-1}$ and the $k_{d}$ was $1.25\times 10^{-2}{\;s}^{-1}$. Compound **10** exhibited a change with a $K_{D}$ of 0.614 μM, where the $k_{a}$ was $2.07\times 10^{4}{\;M}^{-1}s^{-1}$ and the $k_{d}$ was $1.27\times 10^{-2}{\;s}^{-1}$. This showed that compound **10**, with the mixed hotspot interaction pattern, had a higher binding affinity than compound **9**, with one hotspot interaction pattern.

### 3.4. Raw Materials of the Novel Ligands

The raw materials of compounds **9** and **10** were retrieved from an in-house integrated database (Figure 1A). Compound **9** (laetanine) was found in KNApSAcK with C00027405. The raw materials associated with compound **9** were *Lindera glauca* and *Litsea laeta*. *Lindera glauca* extract inhibits proliferation by inducing apoptosis in human colorectal cancer HT-29 cells [62]. Compound **10** (genkwanin) was found in FooDB with FDB006932. The raw materials associated with compound **10** were *Salvia officinalis*, *Thymus vulgaris*, *Salvia rosmarinus*, *Ocimum basilicum*, and *Satureja montana*. *Salvia officinalis* extract showed a potential effect on UV-exposed skin aging [63]. Moreover, *Thymus vulgaris* extract alleviated UVB irradiation by inhibiting MAPK/AP-1 and activating the Nrf2-ARE antioxidant system [64]. The essential oil of *Salvia rosmarinus* showed antioxidant and cholinesterase inhibitory activity [65]. The formulated cream of *Ocimum basilicum* showed anti-aging effects [66]. The potential effects of the raw material extracts are related to the signaling pathways of MMP-9 [67,68,69]. Therefore, we suggest that one of the molecular targets of the raw material extracts from compounds **9** or **10** would be MMP-9, because compounds **9** and **10** directly inhibit MMP-9 with a high binding affinity in this study.

## 4. Discussion

Natural products play a significant role in drug discovery and have two advantages. First, natural products are known to have a high diversity in chemical space [11,70,71]. They are characterized by scaffold diversity and structural complexity and have been considered a major source of oral drugs “beyond Lipinski’s rule of five” [72]. However, since natural products have topological similarities, the overlapping structures may lead to redundant experiments with similar structures. Therefore, we used the BMDMS-NP library, because its structural diversity was comparable to that of commercially available natural products [12]. Secondly, botanical medicines with complex mixtures of natural products have been used because of the potential synergistic therapeutic effects of the components in the mixtures [72]. The search for raw material information from the integrated database can connect a single component to raw material extracts and correlate the inhibitory effects of a single component and extracts. This can support the development of mixtures as therapeutics, such as nutraceuticals and cosmeceuticals.

The FMO method is a useful tool for SBDD because it provides accurate and significant information on the protein–ligand complexes [38,50]. Although the FMO method was successfully applied for a scoring function in this study, the SBVS method requires not only a scoring function but also a docking process. In the comprehensive evaluation of 10 docking programs by Wang et al., the ligand binding poses could be identified in most cases by most docking programs, including Glide [73]. It is important to understand the advantages and limitations of many docking methods before QM methods can be used to predict the binding affinities of the binding poses. Although the docking methods are known to reproduce the binding poses within 2 Å root-mean-square deviation, the small pose generation error may disturb QM-based virtual screening, owing to the high sensitivity of the QM methods to the binding poses. Therefore, it is important to validate the complementarities between QM and docking methods before integrating them into the virtual screening workflow.

In summary, we integrated the FMO method into a virtual screening workflow for quantitative binding affinity prediction and hotspot interaction profiling. FMO analysis with FMO–DFTB3/D/PCM (FMO–TIE) outperformed the Glide docking score and MM-GBSA simulations in the two benchmarking sets. Furthermore, we applied the FMO-based workflow to the hemopexin domain of MMP-9 and proposed two potent natural product inhibitors (laetanin **9** and genkwanin **10**). Laetanin **9** and genkwanin **10**, identified in this study, showed lower migration rates than the control in the wound-healing assays, whereas laetanine **9** and genkwanin **10** directly bind to MMP-9 with a K_D_ of 21.6 and 0.614 μM, respectively. Although laetanine **9** showed a lower migration rate than genkwanin **10**, genkwanin **10** showed a higher inhibition rate in the gelatin zymography assay and bound to MMP-9 more strongly in the SPR assay than laetanine **9**. One of the reasons for this may be the low solubility of genkwanin **10**. More studies to support their functions would be required to use the compounds as active ingredients. Even though the FMO-based virtual screening method was successfully applied to two benchmarking sets and MMP-9, it could be further improved by considering other factors, including desolvation penalty, polarization factors between proteins and ligands, and entropy effects. Therefore, the FMO-based workflow is an efficient SBVS tool for the design, assessment, and filtering of new compounds, which can reduce the effort and cost of chemical synthesis in drug discovery.

## 5. Conclusions

A novel FMO-based virtual screening workflow was introduced to investigate hotspot interaction patterns and to predict virtual hits in SBDD through ab initio quantum mechanical fragment molecular orbital calculations. The FMO-based analysis outperformed the Glide docking score and MM-GBSA simulations as scoring functions for the two benchmarking sets. Through FMO-based virtual screening, we identified two novel natural product inhibitors of MMP-9 (laetanine **9** and genkwanin **10**). Furthermore, we collected raw material information on the natural product inhibitors and correlated the known physiological effects of the raw material extracts with the MMP-9 signaling pathway. Overall, the outcomes of this study can be summarized in two points. First, the promising strategy described in this study could be used in SBDD for targeting MMP-9. Second, the final compounds could be used as active ingredients in nutraceuticals and cosmeceuticals after more studies to support their functions are conducted.

## Data Availability

Not applicable.

## Supplementary Materials

The following supporting information can be downloaded at: https://www.mdpi.com/article/10.3390/ijms23084438/s1.
