# Supplementary material for: Identification of Novel Natural Product Inhibitors against Matrix Metalloproteinase 9 Using Quantum Mechanical Fragment Molecular Orbital-Based Virtual Screening Methods
Source: Int J Mol Sci. 2022 Apr 18;23(8):4438. doi: 10.3390/ijms23084438 (PMC9030947; doi:10.3390/ijms23084438)
Supplement: Supplementary file 1 [file ijms-23-04438-s001.zip › ijms-1662958-supplementary.pdf]

Supporting Information for

Identification of novel natural product inhibitors against matrix metalloproteinase 9 using quantum mechanical fragment molecular orbital-based virtual screening methods

Hocheol Lim [1,2,3,†], Hansol Hong [1,4,†], Seonik Hwang [3], Song Ja Kim [4], Sung Yum Seo [4] and Kyoung Tai No [1,2,5,*]

[1] The Interdisciplinary Graduate Program in Integrative Biotechnology & Translational Medicine, Yonsei University, Incheon, Korea

[2] Bioinformatics and Molecular Design Research Center (BMDRC), Incheon, Republic of Korea

[3] Department of Biotechnology, Yonsei University, Seoul, Korea

[4] Department of Biological Science, Kongju National University, Kongju, Korea

[5] Baobab AiBIO Co., Ltd., Incheon, Korea

[†] Co-first authors

[*] Corresponding author: Kyoung Tai No (ktno@yonsei.ac.kr)

The supporting information for 'Identification of novel natural product inhibitors against matrix metalloproteinase 9 with quantum mechanical fragment molecular orbital-based virtual screening methods' include Figure S1 for the chemical structures, Figure S2-S3 for the FMO results of the six reference ligands, Figure S4 for the 3D pharmacophore model, Figure S5 for the inhibition of the virtual hits in the gelatin zymography assays, Figure S6 for the FMO results of the virtual hits, and Figure S7 for the comparison of hotspot profiles between six references and all ligands in this work.

In this study, there are many abbreviations as follows. AUC, area under the receiver operating characteristic curve; BMDMS-NP, Bioinformatics & Molecular Design Research Center Mass Spectral Library – Natural Products; DFTB, density-functional tight-binding; DFTB3, the third order of density-functional tight-binding; DMEM, Dulbecco's modified Eagle's medium; FBS, fetal bovine serum; FMO, fragment molecular orbitals; HEPES, 4-(2-hydroxyethyl)-1-piperazineethanesulfonic acid; MM/GBSA, molecular mechanics generalized Born surface area; MMPs, matrix metalloproteinase; PCM, polarizable continuum model; PDB, protein data bank; PIE, pair interaction energy; PIEDA, pair interaction energy decomposition analysis; QM, quantum mechanics; RIMP2, the resolution of the identity second-order Møller-Plesset perturbation; ROC, receiver operating characteristics; SAR, structure-activity relationship; SBDD, structure-based drug design; SBVS, structure-based virtual screening; SCF, self-consistent field; SPR, surface plasmon resonance; TIE, total interaction energy.

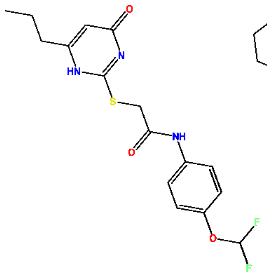
1

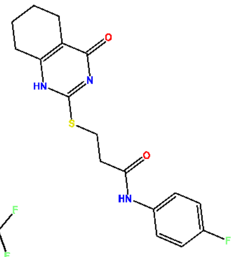
2

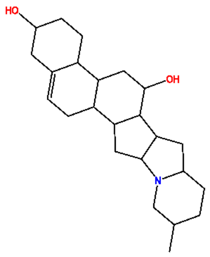
3

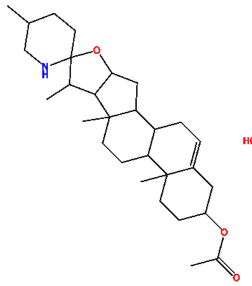
4

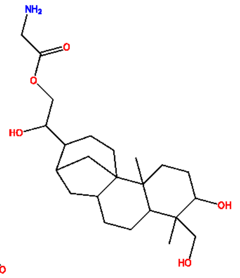
5

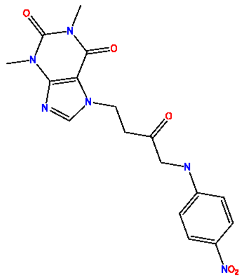
6

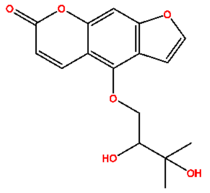
7

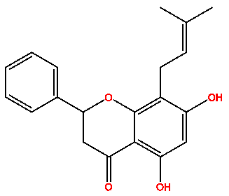
8

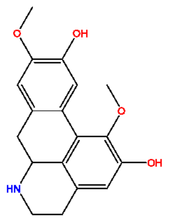
9

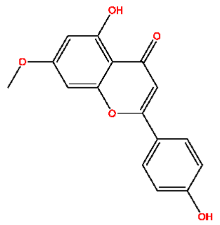
10

Figure S1. Chemical structures of 10 MMP-9/hemopexin inhibitors in this work.

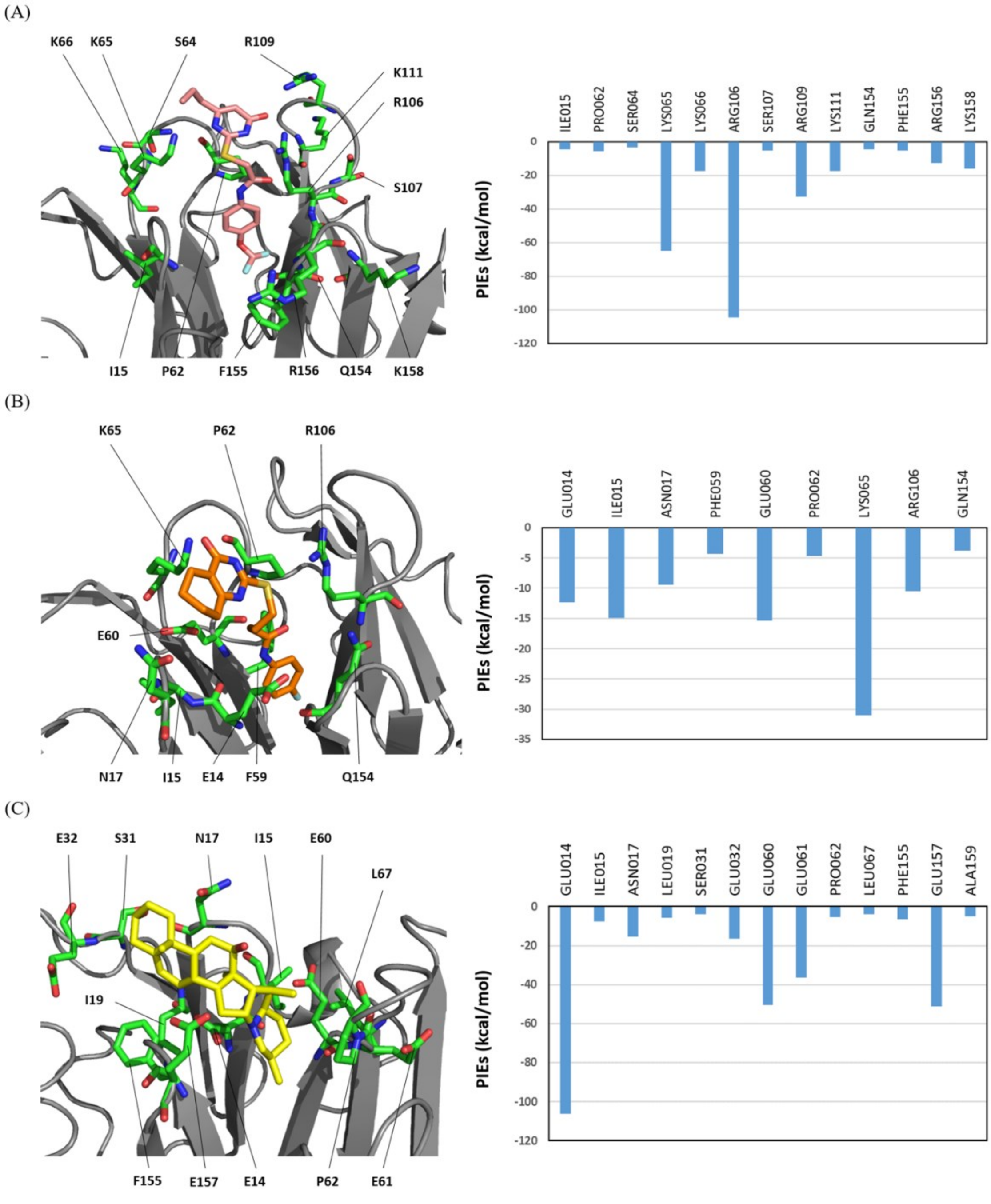

Figure S2. The FMO results for 1, 2, and 3 in complex with hemopexin domain of MMP-9. (**A**) The structure of **1** binding to the hemopexin domain of MMP-9. The carbon atoms of **1** are shown in light red. (**B**) The structure of **2** binding to the hemopexin domain of MMP-9. The carbon atoms of **2** are shown in orange. (**C**) The structure of **3** binding to the hemopexin domain of MMP-9. The carbon atoms of **3** are shown in yellow. The residues of MMP-9 are shown in green. The nitrogen, oxygen, and fluorine atoms are shown in blue, red, and light blue, respectively. The right bar plots describe the PIEs of the significant residues in the hemopexin domain of MMP-9. All interactions shown here have attractive PIE values more stable than -3.0 kcal/mol.

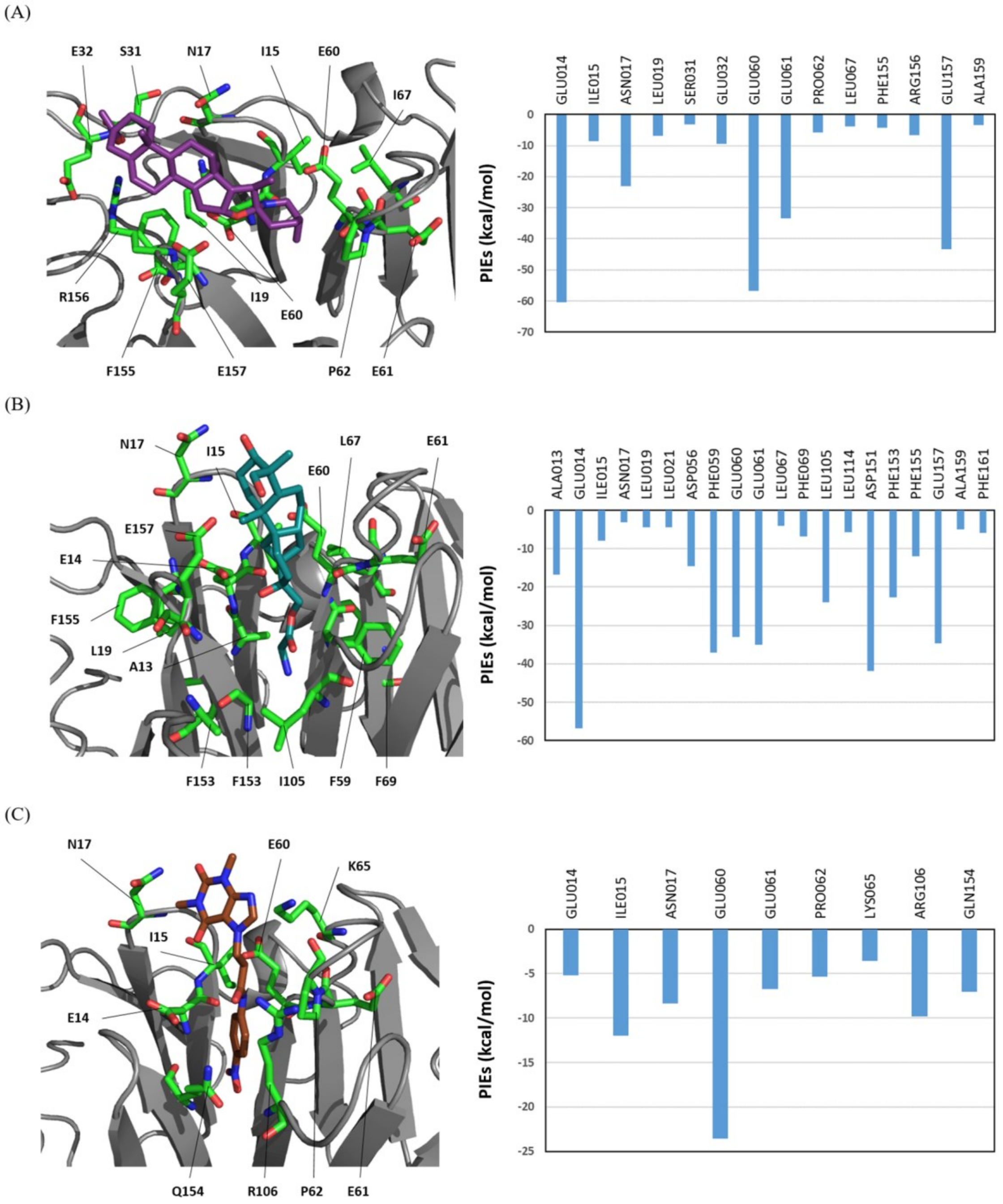

Figure S3. The FMO results for 4, 5, and 6 in complex with hemopexin domain of MMP-9. (**A**) The structure of **4** binding to the hemopexin domain of MMP-9. The carbon atoms of **4** are shown in purple. (**B**) The structure of **5** binding to the hemopexin domain of MMP-9. The carbon atoms of **5** are shown in blue-green. (**C**) The structure of **6** binding to the hemopexin domain of MMP-9. The carbon atoms of **6** are shown in brown. The residues of MMP-9 are shown in green. The nitrogen, oxygen, and fluorine atoms are shown in blue, red, and light blue, respectively. The right bar plots describe the PIEs of the significant residues in the hemopexin domain of MMP-9. All interactions shown here have attractive PIE values more stable than -3.0 kcal/mol.

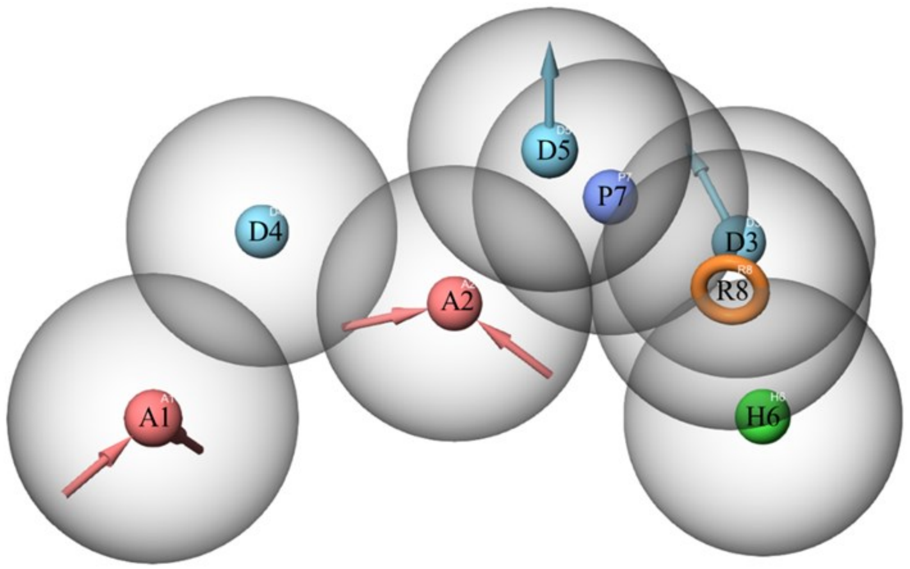

Figure S4. The 3D pharmacophore model of hemopexin inhibitors. The model consists of 8 pharmacophore features, including two hydrogen acceptors (red), three hydrogen donors (cyan), one positive ionic (blue), one hydrophobic (green), and one aromatic ring (orange).

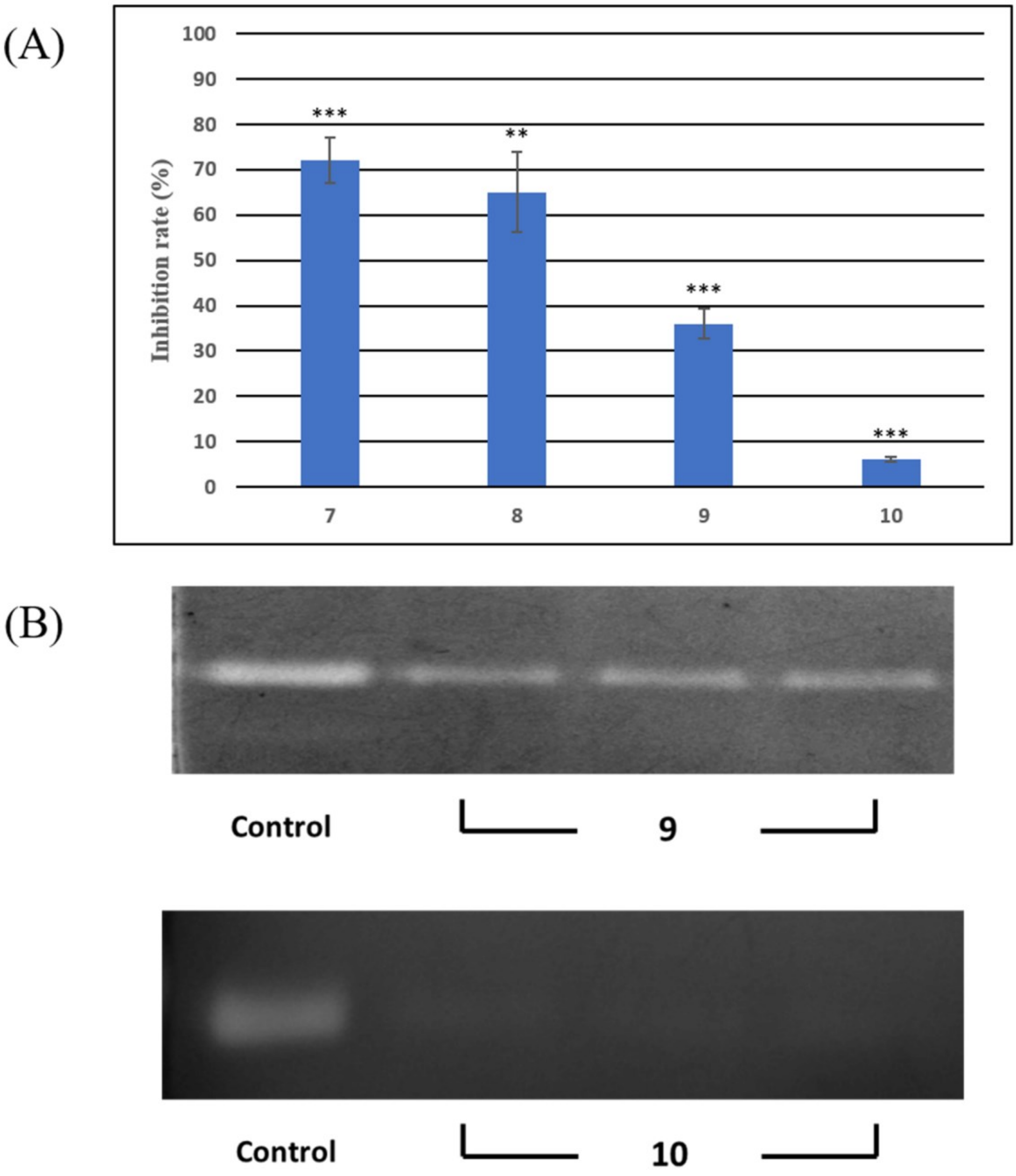

Figure S5. The inhibition of the virtual hits in the gelatin zymography assays. (**A**) The results of the gelatin zymography assays. Columns are the mean percentage of the inhibition rate and the error bars are the standard error of the mean (n = 3). ** $p < 0.01$ and *** $p < 0.001$ vs. control group. (**B**) Gelatin zymography gels of the final hits (**9** and **10**) in this work.

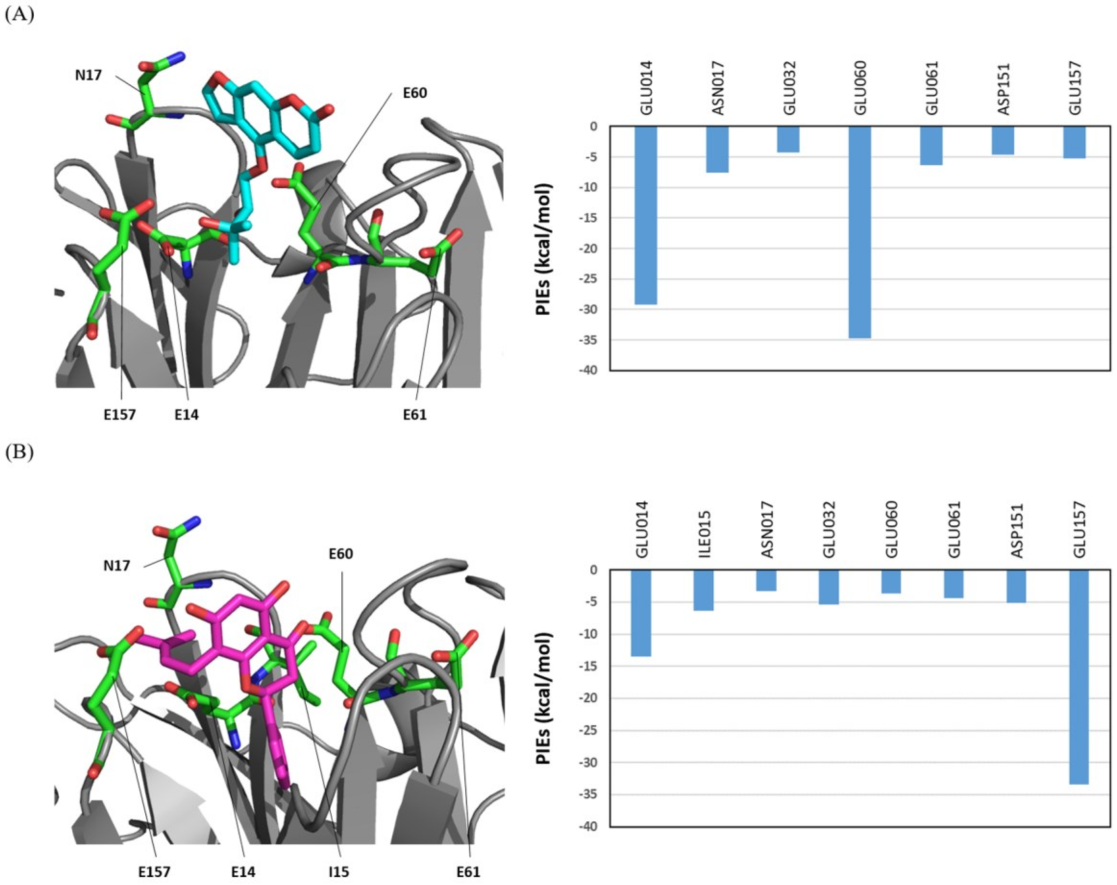

Figure S6. The FMO results for 7 and 8 in complex with hemopexin domain of MMP-9. (**A**) The structure of **7** binding to the hemopexin domain of MMP-9. The carbon atoms of **7** are shown in cyan. (**B**) The structure of **8** binding to the hemopexin domain of MMP-9. The carbon atoms of **8** are shown in magenta. The residues of MMP-9 are shown in green. The nitrogen, oxygen, and fluorine atoms are shown in blue, red, and light blue, respectively. The right bar plots describe the PIEs of the significant residues in the hemopexin domain of MMP-9. All interactions shown here have attractive PIE values more stable than -3.0 kcal/mol.

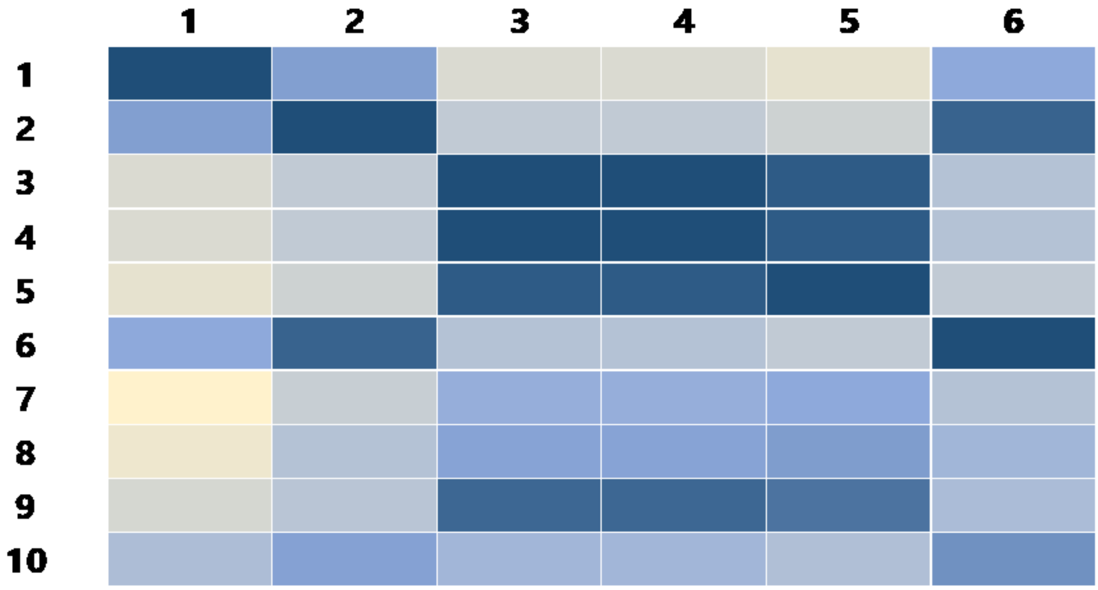

Figure S7. Comparison of hotspot profiles among ligands. Protein-ligand information is shown in the columns (reference) and rows (all ligands). The similarities of hotspot profiles of ligands are measured with Tanimoto fingerprint generated from the FMO analysis at FMO-RIMP2/PCM level. In each box of the matrix, the similarity values of hotspot profiles are colored from dark blue (1.0), blue (0.5), to light-yellow (0.0).
